# Spiking a Silty-Sand Reference Soil with Bacterial DNA: Limits and Pitfalls in the Discrimination of Live and Dead Cells When Applying Ethidium Monoazide (EMA) Treatment

**DOI:** 10.1007/s00284-019-01772-y

**Published:** 2019-09-24

**Authors:** Andreas O. Wagner, Nadine Praeg, Paul Illmer

**Affiliations:** Department of Microbiology, Universität Innsbruck, Technikerstr. 25d, 6020 Innsbruck, Austria

## Abstract

In the present study, EMA (ethidium monoazide) treatment was applied to a silty-sand reference soil prior to DNA extraction to enable a differentiation between dead and living cells. For this purpose, a reference soil was spiked with *Listeria monocytogenes* cells or cell equivalents, respectively. With the purpose of evaluating optimum treatment conditions, different EMA concentrations have been tested. However, the results remained largely inconclusive. Furthermore, varied dark incubation periods allowing EMA to penetrate dead cells did not allow the selective removal of DNA from membrane-compromised cells in downstream analyses. In contrast to undiluted soil, an effect of EMA treatment during DNA extraction could be observed when using a 1:10 dilution of the reference soil; however, the effect has not been sufficiently selective to act on heat-treated cells only. Although the application of EMA to soil requires further evaluation, the procedure harbors future potential for improving DNA-based approaches in microbial ecology studies.

## Introduction

Polymerase chain reaction (PCR) has progressively developed to become an indispensable technique in medical and biological applications as it allows the amplification of any DNA fragment present in a certain sample. Despite the many advantages, data interpretation of PCR-based approaches in microbial ecology is still hampered by lacking discrimination between DNA originating from intact, alive cells and dead, fragmented cell deposits. In particular, the extraction of the requested intracellular DNA from environmental samples is biased by co-extraction of extracellular nucleic acids that may considerably contribute to the total DNA yield, and thus overestimate the number of viable cells in a certain habitat [1–3]. However, for a reliable (and exact) determination of microbial communities—regarding both, diversity and abundance—it is of particular interest to specifically target the living population when employing molecular biological, DNA-based methods in microbial ecology studies. Methodical progress remains essential to optimize DNA extraction and amplification efficiencies to avoid false-positive signals [4]. Otherwise, RNA-based approaches, which would target the active part of a microbial community, can be problematic due to the fast RNA-decay rates after loss of cell viability, are more expensive, laborious, and error-prone with respect to DNA-based analysis, and experience difficulties as well as often need extensive protocol adaptation when extracted from biosolids [5–8].

A rather novel technique employs the addition of intercalating dyes including ethidium monoazide (EMA) or propidium monoazide (PMA) during DNA extraction to enable the differentiation between live and dead cells in subsequent molecular biological analyses. This technique has been extensively used for qPCR applications to mask DNA from dead, membrane-compromised cells [9–16]. It has proven to be successful with pathogen-related samples [17–23] and is promising to study environmental samples [24–34]. The procedure involves the application of EMA or PMA, followed by the extraction of genomic DNA and subsequent PCR amplification. Prior to conventional DNA extraction, the intercalating dye is added to the desired extraction matrix that covalently binds to genomic DNA by conversion of the azide group into a nitrene radical upon photolysis [35]. As a consequence, DNA extraction and PCR amplification of these fragments is restricted [12, 27, 36]. Since EMA and PMA are hypothesized not to be able to penetrate cytoplasmic membranes [37], free DNA or DNA derived from microorganisms not maintaining cell wall integrity is selectively bound during extraction. Hence, the active (viable) part of the microbial community of a certain habitat can be targeted. Although both dyes share similar characteristics as intercalating dyes, they differ in respect of their permeation behavior. Due to its chemical composition, EMA is more efficient in signal suppression and equally effective on Gram-positive and Gram-negative bacteria [38]; however, compared with PMA it is less selective and could underestimate the viable population [32, 39]. Recent studies also investigated a mixture of both dyes [40, 41]. With a few exceptions, this technique has rarely been used for microbial diversity and/or abundance characterization of environmental samples and an investigation of the possible scope of application is still missing for many habitats including soil.

The present study therefore aimed at evaluating the overall applicability of EMA (Phenanthridium, 3-amino-8-azido-5-ethyl-6-phenyl, bromide) treatment to selectively target and mask DNA from cell-compromised *Listeria monocytogenes* in an Eutric Flavisol reference soil.

## Material and Methods

### Reference Soil

The present study used a reference soil derived from Landwirtschaftliche Untersuchungs- und Forschungsanstalt Speyer, Germany (https://www.lufa-speyer.de/). It is defined as “Reference Soil 2.3” for soil type silty sand (uS) (after German DIN) or sandy loam (after U.S. Department of Agriculture (USDA)). Based on the “World Reference Base for Soil Resources 2014” (FAO, 2015), it is classified as Eutric Flavisol and was also used for previous investigations [36]. Basic soil characteristics can be found in Table 1.

**Table 1:** Basic soil characteristics for the used reference soil

Parameter	Mean (± SD)
Soil type^a^	Silty sand (uS) (DIN), sandy loam (USDA)
pH (in 0.01 M CaCl_2_)^a^	5.84 (0.6)
Organic carbon [g/100 g]^a^	0.68 (0.04)
Nitrogen [g/100 g]^a^	0.08 (0.01)
Max. water holding capacity [g/100 g]^a^	35.4 (1.0)
Weight per volume [g/1000 ml]^a^	1307 (41)
Total extractable DNA [µg DNA g^−1^ DW]^b^	3.30 (0.606)

All values refer to dry matter

^a^According to LUFA, Speyer

^b^*n* = 12, single extraction

### Experimental Design and Setup

In order to investigate the applicability of EMA pretreatment for DNA extraction of vital cells only in soil, a series of experiments was conducted. These had in common that DNA derived from *Listeria monocytogenes* was added in known concentrations to soil either as intact or heat-treated cells (cell equivalents, heat-treated cells). Subsequently, DNA extraction was performed with (EMA-DNA extraction) and without EMA pretreatment (refer to 2.5 and 2.6) followed by the quantification of the DNA extracts and/or listeriolysin O gene copy numbers via qPCR assays (refer to 2.7). All experiments using soil as a matrix were carried out at least in triplicate.

As an initial step, and before using soil as starting material, the successful removal of DNA derived from damaged cells using EMA was evaluated in a non-soil environment. Serial dilutions in PBS buffer from 1 × 10^9^ down to 1 × 10^3^ cells ml^−1^ were subjected to DNA extraction with and without an additional EMA pretreatment step. Equivalent amounts of heat-treated cells served as controls. EMA treatment was carried out using a concentration of 50 µg ml^−1^ according to previously published recommendations [13, 36] under identical dark incubation and light activation conditions as described in [27] (please also refer to 2.5). This analysis was carried out in duplicate for EMA- and non-EMA-treated samples, respectively.

In a second step, parameter variation studies including EMA concentrations and dark incubation periods aimed at finding a protocol for the EMA-DNA extraction from soil. To evaluate the impact of initial EMA concentrations (0, 66, 100, 250, and 500 µg EMA g^−1^ soil) the reference soil was spiked with 1 × 10^8^ ml^−1^ intact (C) or heat-inactivated, disrupted cells (D), respectively (*n* = 70).

Optimum dark incubation conditions during EMA treatment were investigated by adding 100 µg EMA g^−1^ soil (*n* = 28) under varied time periods spanning 20, 60, and 90 min. As a control, DNA extracts from soil containing 1 × 10^8^ cells or cell equivalents per gram, respectively, were evaluated without EMA treatment.

Furthermore, in sterile distilled water a 1:10 diluted solution of the reference soil (0.5 g fresh weight) was prepared in triplicates and spiked with intact and heat-treated cells, respectively. Thus, different mixtures of cells and cell equivalents in one sample (*n* = 24) (Table 2) were resulting and the effect of EMA pretreatment on heat-treated cells in the presence of intact cells was evaluated. An aliquot of diluted soil served as a control; spiked soil dilutions as well as controls were treated in the same way. The prepared suspension was shaken for 15 min at 125 rpm and 25 °C.

**Table 2:** Composition of samples containing different concentrations of intact (C) and heat-treated, disrupted (D) cells in 1:10 diluted soil and CFU count as well as obtained listeriolysin O copy numbers with and without EMA pretreatment during DNA extraction

Intact cell spike (log)	Heat-treated cell spike (log)	EMA treatment	Abbrev	CFU count [log] (± SD)	Obtained copy number [log] (± SD)	Expected copy number (log)	Expected-obtained [log]
5 × 10^8^ (8.7)	1 × 10^5^ (5.0)	Yes	C8D5 +	8.61 (0.12)	6.36 (0.96)	8.7	2.34
1 × 10^7^ (7.0)	1 × 10^7^ (7.0)	Yes	C7D7 +	7.35 (0.04)	6.49 (1.26)	7.0	0.51
1 × 10^5^ (5.0)	5 × 10^8^ (8.6)	Yes	C5D8 +	5.26 (0.05)	6.82 (0.10)	5.0	− 1.82
1 × 10^5^ (5.0)	1 × 10^5^ (5.0)	Yes	C5D5 +	5.19 (0.08)	6.83 (0.96)	5.0	− 1.83
5 × 10^8^ (8.7)	1 × 10^5^ (5.0)	No	C8D5	8.78 (0.08)	9.67 (0.31)	8.7	− 0.97
1 × 10^7^ (7.0)	1 × 10^7^ (7.0)	No	C7D7	7.40 (0.19)	7.18 (0.74)	7.3	0.12
1 × 10^5^ (5.0)	5 × 10^8^ (8.6)	No	C5D8	5.31 (0.05)	8.68 (0.47)	8.7	0.02
1 × 10^5^ (5.0)	1 × 10^5^ (5.0)	No	C5D5	5.22 (0.10)	6.16 (0.25)	5.3	− 0.86

In a last experiment, a soil dilution was prepared in PBS buffer supplemented with 5 mM EDTA (PBS-EDTA) in order to minimize possibly interacting of the soil matrix with EMA treatment (*n* = 18). For this purpose, three 50-ml tubes were filled with 1.0 g soil; 1 × 10^8^ cells were added (suspended in PBS-EDTA), as well as 1 × 10^9^, 1 × 10^8^, and 1 × 10^7^ cell equivalents, respectively, and filled up with PBS-EDTA to a final volume of 10 ml in order to achieve a mixture of 1 × 10^7^ cells plus 1 × 10^8^, 1 × 10^7^, and 1 × 10^6^ cell equivalents per ml, respectively. The prepared suspensions were shaken for 15 min at 125 rpm and 25 °C, with 1.0 ml of the liquid phase being subsequently used for DNA extraction in three replicates.

### Cultivation of *L. monocytogenes*

*Listeria monocytogenes* (DSM 15675) was grown in DSM medium 92 containing 30.0 g Trypticase soy broth, 3.0 g yeast extract, and 1000 ml of distilled water (pH 7.0). Cells were harvested after 24–30 h of incubation by centrifugation (7000×*g*, 10 min), washed twice (¼ Ringer solution or PBS-EDTA), and re-suspended in ¼ Ringer solution or PBS-EDTA. Freshly prepared suspensions were either used directly for spiking or underwent a heat treatment (refer to 2.4). Cell counting was performed using a Thoma-chamber (0.0025 mm^2^ × 0.01 mm) after dilution in Ringer solution. CFU numbers were determined by preparing serial dilutions and plating 50 µl on DSM medium 92 agar dishes.

### Heat Treatment

Cell suspensions (0.75 ml, in ¼ Ringer solution) were filled into micro-centrifuge tubes and heated for 5 min at 95 °C in a water bath as described before [33]. Aliquots were then plated on DSM medium 92, incubated for 72 h at 37 °C, and checked for microbial growth. Remaining samples were stored in low DNA binding tubes (Biozym, Germany) at − 20 °C until further processing.

### EMA Treatment

EMA pretreatment for soil samples was started by mixing 250 mg soil with 250 µl of molecular grade water to the extraction tubes containing the soil samples. After the addition of EMA (Biotium), the tubes were vortexed for 10 s to secure a homogenous distribution of EMA. Subsequently, the samples were incubated in the dark for 10 min (non-soil samples) and 45 min (soil samples) allowing EMA to penetrate membrane-compromised cells. Then, the samples were exposed to light by placing them 10 cm away from a 650-W halogen device. In previous investigations using non-environmental samples, light activation periods ranged from 60 s [9, 34], to 5 min [17], and up to 20 min [19]. In the present study, light activation in light transparent tubes (Biozym, Germany) was performed for 20 min to secure the best possible covalent binding of EMA to DNA, since turbidity can impede the efficiency of EMA treatment using environmental samples [42]. To avoid heating of the samples, light activation was carried out on ice, which had to be exchanged in 10-min intervals. Samples were mixed very gently every 5 min. Following the treatment with EMA, DNA extraction was conducted according to “DNA Extraction and Quantification” section starting with the addition of buffer and the beat-beating step. Control samples (without EMA treatment) were handled analogously (dark incubation and light activation) but without EMA addition.

For EMA pretreatment of 1:10 soil extracts 0.5 ml (extract in water) or 1.0 ml (extract in PBS-EDTA) was transferred into DNA extraction kit’s beat-beating tubes. Subsequently, EMA (100 µg EMA ml^−1^ extract) was added and a dark incubation conducted for 10 min followed by 15-min light activation time. Samples were centrifuged for 5 min at 12,000×*g*. For the PBS-EDTA extracts, 250 µl of supernatant were discarded and taken into account when calculating copy numbers. Posterior DNA extraction steps were conducted as described in “DNA Extraction and Quantification.”

### DNA Extraction and Quantification

As the extraction of DNA from soil can be critical, the procedure adopted here was optimized in a previous investigation [36]. DNA was extracted using the Soil Extract II Kit (Macherey & Nagel, Germany) following the recommendation of the manufacturer, except that the beat-beating step was done twice on a FastPrep 24-5G (MPbio) with an interval of 300 s. Subsequently, the quantity and quality of the extracted DNA was evaluated fluorimetrically with PicoGreen dsDNA quantification reagent (Invitrogen, Carlsbad, USA, Anthos-Zenyth Multimode Detector) and spectrophotometrically with NanoDrop 2000c™ (PEQLAB, Germany) (Abs@260 nm = 1.0 referred to a concentration of 50 ng µl^−1^), respectively.

### qPCR

qPCR was performed to quantify the listeriolysin O gene (hly A) in DNA extracts, which further allowed for the indirect estimation of *Listeria monocytogenes* abundance in spiked soils. A method proposed by [43] was used and qPCR performed on a Corbett Life Science (Qiagen) Rotor-Gene 6000 system. Prior to experiments, the primers and reaction conditions were validated with DNA extracts of a known number of *L. monocytogenes* cells. All PCR reactions and approaches were performed at least in duplicates, with “non-template controls” (NTCs) and positive controls using 2 µl of template DNA in a final volume of 20 µl being applied. Template DNA was diluted to achieve total DNA concentrations of 0.5–5 ng µl^−1^ prior addition to the reaction mix (at least 1:10) and taken into account in the calculation of copy numbers. As a standard, the purified PCR product targeting the hly A gene associated with DNA from a pure culture of *Listeria monocytogenes* was used in known concentrations as described before for the quantification of Archaea [44]. For quantification and efficiency calculations, diluted standards were used and the CT (cycle threshold) values were plotted against the log of given templates to obtain standard graphs as described in [45]. qPCR data were used when an efficiency value of at least 0.9 (90%) was reached. Standard curves resulted in *R*^2^ between 0.999 and 0.947. The efficiency analysis of the qPCR reactions was calculated by the Rotor-Gene software.

### Statistical Analysis

Statistical analysis was performed by using the Software package *Statistica 9.0* (StatSoft^®^) and *SigmaPlot 12.0* (Systat Software Inc.). Results are given as means ± standard deviation. Significant differences were ascertained by one-way or multifactorial ANOVA. A significance level of 0.05 was used to assess differences between treatments. *Bonferroni Test* was used to discriminate between single variants.

## Results and Discussion

### EMA Application in a Non-soil Environment

To validate the previously shown potential of EMA to selectively bind to DNA from cells not maintaining cell wall integrity [9, 13, 46], serial dilutions of *Listeria monocytogenes* cultures or equivalent amounts of heat-treated cells (1 × 10^9^ to 1 × 10^3^ ml^−1^) were used in a non-soil environment. Prepared dilutions were either subjected to EMA treatment or not, followed by DNA extraction and subsequent determination of the copy number of hyl A gene by qPCR. Although previous studies used lower concentrations of intercalating dyes [47] or demonstrated a positive effect of EMA if applied in much lower concentrations [17, 33], we used 50 µg EMA ml^−1^. This was taking into account the given high number of desired 1 × 10^9^ cells ml^−1^, which corresponds to an approximate amount of 5 µg of total DNA when calculating 5 fg DNA per cell [48].

qPCR results for the desired amount of 1 × 10^9^ to 1 × 10^3^ copies ml^−1^ showed higher copy numbers (14–38%) for samples with intact cells than for those with equivalent amounts of heat-inactivated cells. While the number for intact cell samples remained in the expected range, the copy number obtained for heat-treated cells significantly ceased when EMA treatment was applied. From an initial concentration of 2.84 × 10^9^ (as measured by qPCR in the sample containing intact cells), a reduction to 5.92 × 10^5^ copies ml^−1^ could be achieved. Although the complete portion of cracked cell’s DNA could not be eliminated by EMA treatment, at least a reduction of approx. 4 log_10_ could be attained. For samples containing a spike of 1.08 × 10^7^, 7.66 × 10^4^, and 3.09 × 10^3^ heat-treated cells (copy numbers obtained from samples without EMA treatment), at least one of two replicates resulted in a qPCR result below the detection limit (no template control, NTC), while the remaining ones were very close to NTC. Thus, efficient binding of EMA to the DNA present in the samples for samples containing < 1.08 × 10^7^ heat-treated cells was assumed as also observed in other studies [13]. In conclusion, preliminary results in a non-soil matrix were promising and clearly indicated a high potential of EMA treatment. Subsequent experiments aimed at the investigation of EMA treatment and the removal of DNA from non-vital cells in a reference soil.

### EMA Application in Soil Environment

To evaluate the potential of EMA treatment prior DNA extraction from soil, the same parameters as for the non-soil environment were applied; however, instead of using 1 × 10^9^ cells or cell equivalents (heat-treated cells) 1 × 10^8^ cells were used in order to decrease the total amount of DNA that is addressed to EMA binding. However, it became obvious that the treatment parameters (EMA concentration, dark incubation, light activation) were not appropriate to see a reduction in hyl A copy numbers via qPCR. Melt curve analysis did not reveal any differences in melting peaks between EMA-treated and untreated samples, respectively, so the poor effect was attributable to the insufficient action of EMA.

**Fig. 1:**
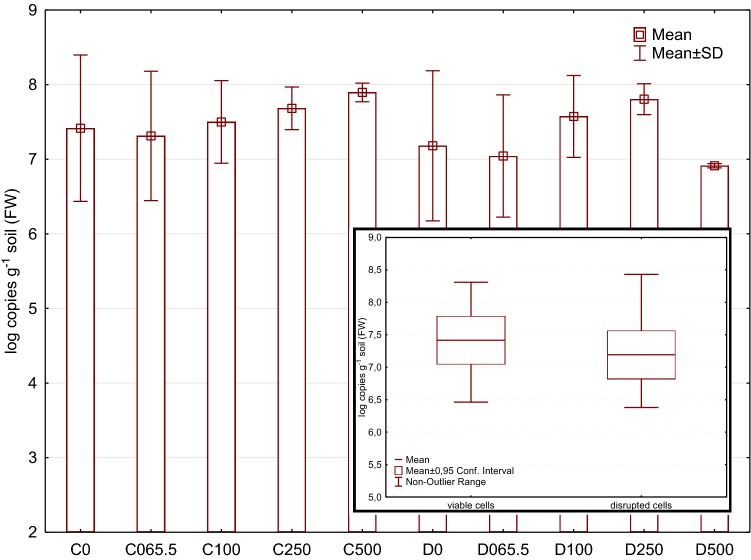
Copy number (qPCR) for different EMA concentrations (0, 65.5, 100, 250, 500) applied prior DNA extraction from a reference soil spiked with 1 × 10^8^ viable cells (C) or an equivalent amount of heat-treated, disrupted (D) cells. Inset: box plot calculated from all EMA-treated C and D samples showing no significant difference (ANOVA, *p* < 0.05). Numbers reflect the applied EMA concentrations (µg g^−1^ soil); C0 and D0 the co-processed controls without EMA addition

So in a next step different EMA concentrations were tested. Results are depicted in Fig. 1. Even though concentrations of up to 500 µg EMA g^−1^ soil were applied during EMA treatment, a significant effect could not be verified. Although the results indicated sufficient resolution of qPCR and demonstrated that the comparability of results for intact and heat-treated cell was given even in the soil environment (Fig. 1, samples lacking EMA treatment C0 and D0), EMA treatment did not result in the expected decrease in target DNA from samples containing heat-treated cells. Possible explanations include a too high number of target molecules [49] as either the concentration of EMA was still too low or the binding of EMA was insufficient during dark incubation. Moreover, the interaction of EMA with soil particles might have been responsible for a reduced effectivity of EMA since the high content of silt and sand together with a substantial amount of clay reflect very large surface areas. This possibly led to an absorbtion of EMA as well as to problems with the light activation due to the matrix hampering the covalent binding of EMA [27]. However, also various other environmental factors are known to negatively influence the binding of EMA to DNA [17].

**Fig. 2:**
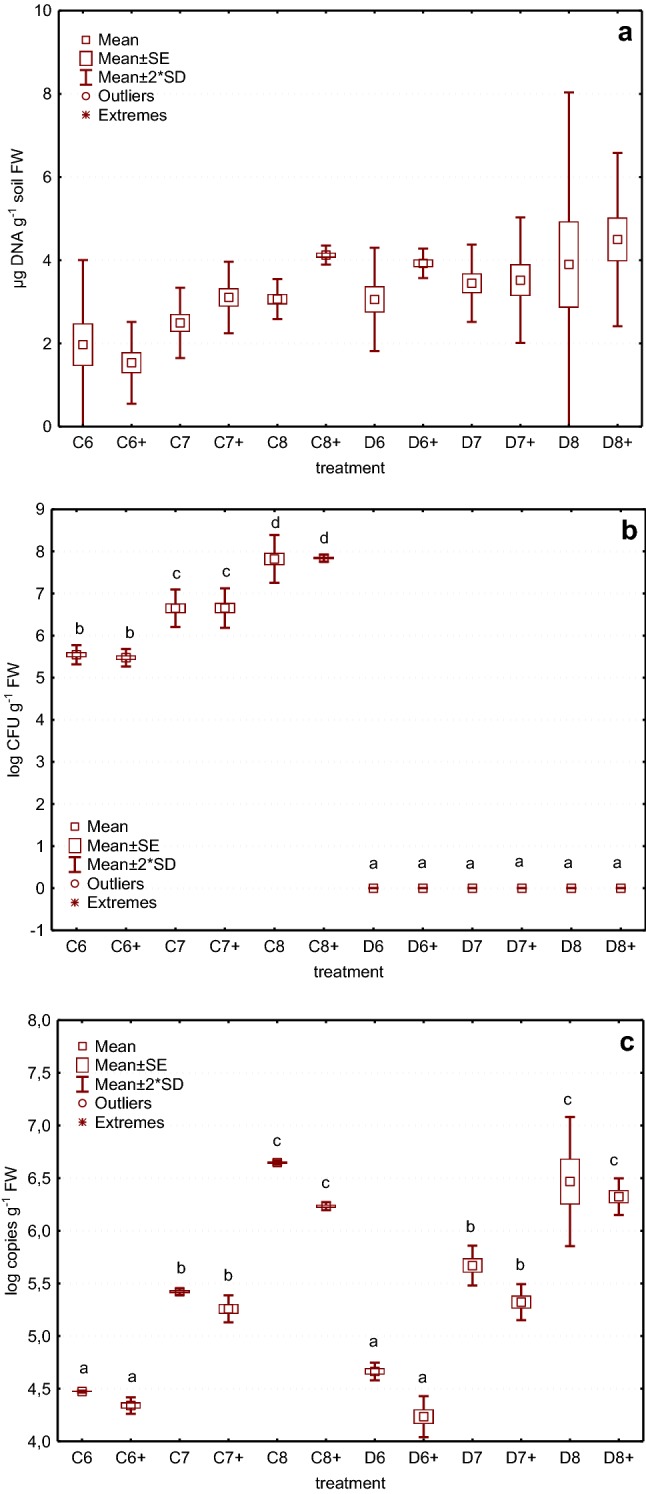
Total DNA concentration (**a**), CFU count (**b**), and hly A gene copy number (**c**) for a reference soil spiked with 1 × 10^6^, 1 × 10^7^, or 1 × 10^8^ cells (C) or an equivalent amount of heat-treated, disrupted (D) cells. Samples were either subjected to an EMA treatment (+) (100 µg g^−1^ soil FW) or not. Significant differences are indicated by different characters (*p* < 0.05, Bonferroni post hoc test)

The number of target DNA from cells not maintaining cell wall integrity was varied by adding 1 × 10^6^, 1 × 10^7^, and 1 × 10^8^ cells or equivalents g^−1^ soil (FW) (Fig. 2); however, a significant impact of EMA treatment on total DNA yield between soil spiked with intact and heat-treated cells, respectively, was missing (Fig. 2a). On the contrary, the addition of viable cells became clearly apparent by classical cultivation, although CFU counts were slightly lower than the calculated spike. Furthermore, in samples containing heat-treated cells, no living cells could be verified (Fig. 2b). While data for total DNA lacked significant differences between the spiking variations, qPCR amplification of the listeriolysin gene allowed a clear differentiation but underestimated the amount of added cells and cell equivalents considerably. A significant effect of EMA treatment to mask DNA from heat-treated cells and protect them from being amplified during qPCR could not be observed, although a general trend of higher Ct values of EMA-treated samples was found irrespective of the addition of cells or heat-treated cell equivalents (Fig. 2c). In conclusion, an increase of EMA concentration up to 100 µg g^−1^ soil (FW) and a concurrent reduction of target DNA from 1 × 10^9^ to max. 1 × 10^8^ did not result in the desired reduction of extractable DNA from heat-treated cells in the applied soil as determined by qPCR measurement and led to clearly false-positive signals in EMA-treated samples.

We thus hypothesized that the soil matrix or its composition reduced the effect of EMA treatment. To test the impact of the amount of extracted soil on DNA extraction in combination with EMA, the initial weight of soil used for DNA extraction was reduced. For this experiment, 50 or 100 mg of soil (instead of 250 mg) spiked with 1 × 10^8^ cells or equivalents of *L. monocytogenes* was treated with 25 µg EMA and used for DNA extraction. The effect, however, was statistically negligible and resulted in 4.55 × 10^7^ and 8.37 × 10^7^ copies g^−1^ soil after qPCR for EMA-treated and non-treated samples, respectively.

Besides testing different EMA concentrations, the impact of dark incubation periods was also evaluated. Previously described procedures applied time periods ranging from 5 [9, 34], to 10 [17, 50], and up to 60 min [19]. In the present study, dark incubation periods extending over 20, 60, and 90 min were applied allowing EMA to penetrate membranes of cells not maintaining cell wall integrity prior to light activation. While the controls without EMA treatment showed copy numbers in the expected range of 1 × 10^8^ copies g^−1^ soil for intact as well as for heat-treated cells, copy numbers for samples with intact cells were slightly higher. However, EMA treatment in combination with 20 min of dark incubation did not result in significant differences between samples containing intact and heat-treated cells, respectively. Even a prolongation of dark incubation during EMA treatment did not result in a significant reduction of copy numbers obtained from samples containing heat-treated cells and samples with intact cells. For a dark incubation time during EMA treatment of 60 min, 1.34 × 10^7^ and for 90 min 3.83 × 10^7^ copies were found.

In a last step, 1:10 soil extracts were prepared, first in distilled water and then in PBS-EDTA. The water soil extract was spiked with different concentrations of *L. monocytogenes* cells or equivalents (5 × 10^8^, 1 × 10^7^, 1 × 10^5^) and EMA treatment was tested. For this experiment, a combination of intact cells and heat-treated cells was used according to Table 2. Negative controls were tested several times before using DNA extracts from soil without adding any target DNA that resulted in Ct values within the range of the no template control during qPCR analysis (data not shown). Then, the effect of EMA treatment for DNA from heat-treated cells was evaluated via qPCR in the presence of target DNA from intact cells.

**Fig. 3:**
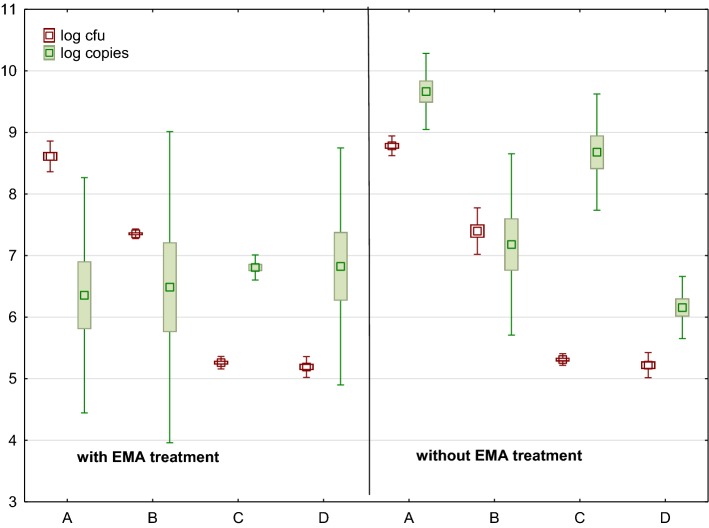
CFU and copy number per ml of soil extract (Mean, Box: Mean ± SE; Whisker: Mean ± 2*SD). The extract was spiked with A: 5 × 10^8^ cells and 1 × 10^5^ heat-treated cells, B: 1 × 10^7^ cells and 1 × 10^7^ heat-treated cells, C: 1 × 10^5^ cells and 5 × 10^8^ heat-treated cells, and D: 1 × 10^5^ cells and 1 × 10^5^ heat-treated cells simultaneously. Please also refer to Table 2

In contrast to the results obtained from DNA extracted directly from soil, qPCR results from 1:10 diluted soil samples showed an impact of EMA treatment. However, the desired effect to selectively and exclusively mask DNA from heat-treated cells could further not be verified (Table 2, Fig. 3). While CFU counts represented spiked cell concentrations fairly well, the effectiveness of EMA failed and did not eliminate DNA from compromised cells sufficiently (Fig. 3). Obviously, the action of EMA was unspecific also targeting intact cells and ineffective when using high numbers of heat-treated cells in diluted soil samples. Besides, applying EMA treatment to both cells and cell equivalents resulted in similar log_10_ copy numbers between 6.36 and 6.83, although a difference of 3 log_10_ between samples containing the highest and lowest amount of intact cells was expected (Table 2). Therefore, an underestimation of the real copy numbers was the consequence, an effect that has also been described earlier [37, 38]. On the other hand, an overestimation of copy number could be observed when using diluted soil samples without EMA treatment. This could be related to the additional incubation time during preparation of dilutions, which was further reflected in a slight increase in the number of CFU counts.

**Fig. 4:**
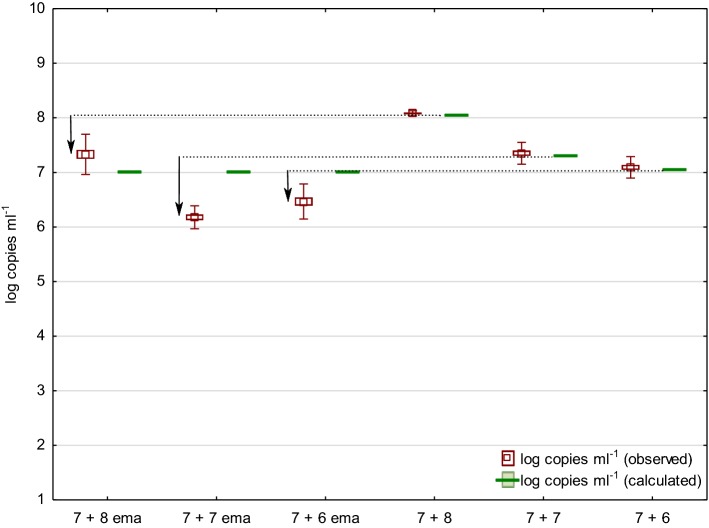
Copy number per ml of PBS-EDTA soil extract (Mean, Box: Mean ± SE; Whisker: Mean ± 2*SD, green lines refer to calculated copy numbers). The extract was spiked with 1 × 10^7^ cells and 1 × 10^8^ (7 + 8), 1 × 10^7^ (7 + 7) and 1 × 10^6^ (7 + 6) heat-treated cells per ml, respectively. The samples were treated with (EMA) and without EMA, arrows indicate the reduction of copy numbers by EMA

These results implied a still insufficient action (specificity and effectivity) of EMA possibly due to interaction of EMA with the soil matrix. Thus, an additional dilution was prepared in PBS-EDTA comprised of 1 × 10^7^ intact cells and 1 × 10^8^, 1 × 10^7^, and 1 × 10^6^ cell equivalents, respectively. Results are depicted in Fig. 4. Via the utilization of an EDTA containing buffer, the effectivity of EMA could be increased and the non-desired effect on intact cells was reduced. Although for example the copy number of 1 × 10^7^ intact cells was overestimated by a factor of 2.28 when 1 × 10^8^ heat-treated cells were present, this indicates that DNA from approx. 7.7 × 10^7^ cell equivalents were masked by EMA and excluded from qPCR amplification (Fig. 4). However, an impact of EMA on vital cells was still observed and led to underestimation of the real number of vital cells (Fig. 4).

Generally speaking, the application of EMA directly to a silty-sand reference soil did not enable a clear differentiation between DNA from cell wall-compromised and living cells, respectively. Although DNA extraction from spiked soil is routine now it remains a challenging technique per se [51–53]. In the present investigation, qPCR resulted in copy numbers reflecting the dimension of the spiking and thus indicated proper DNA extraction (efficiency) and accurate qPCR amplification. Nevertheless, the application of different EMA concentrations led to inconclusive results. Varying periods of dark incubation to permit the passage of EMA into the compromised cells did not allow the complete exclusion of DNA from cells not maintaining cell wall integrity in downstream analysis. Lacking effectivity of EMA treatment can be associated with the matrix it was applied to, since complex matrices can adversely influence the efficiency of EMA treatment [49, 54]. The high content of silt and sand together with a substantial amount of approx. 10% clay reflect very large surface areas, which might have been able to absorb concentrations of up to 500 mg EMA g^−1^ soil. Other parameters known to limit the success of EMA treatment include high salt concentrations, pH, presence of high amounts of dead cells, and turbid samples [27, 49].

In contrast to direct application, an effect of EMA treatment during DNA extraction could be observed when using a 1:10 dilution of the reference soil in water or PBS-EDTA. By applying soil dilutions (instead of direct application), the effect of EMA on high target numbers (1 × 10^8^ cell equivalents) could be strengthened und resulted in a copy number close to the calculated ones. However, by the applied EMA-DNA extraction procedure still a portion of non-target molecules was masked by EMA.

Insights gained in the present study, especially the use of soil dilutions applying PBS-EDTA buffer prior to DNA extraction, might serve as a basis for subsequent investigations. Suggested improvements further include the application of primers generating longer fragments as a solution to avoid false-positive signals [18, 55, 56]. However, promising results of removing DNA from non-viable cells via intercalating dyes in other habitats [29, 40, 41, 57–60] give courage for the applicability of this methodology for soil habitats in the future.
